# Rice Bran Arabinoxylan Compound and Quality of Life (RBAC-QoL) of Cancer Patients: An Interim Analysis of the RBAC-QoL Study

**DOI:** 10.7759/cureus.53188

**Published:** 2024-01-29

**Authors:** Soo Liang Ooi, Peter S Micalos, Rob Zielinski, Sok Cheon Pak

**Affiliations:** 1 Integrative/Complementary Medicine, School of Dentistry and Medical Sciences, Charles Sturt University, Bathurst, AUS; 2 Anatomy and Physiology, School of Dentistry and Medical Sciences, Charles Sturt University, Port Macquarie, AUS; 3 Oncology, Central West Cancer Centre, Orange Health Service, Orange, AUS; 4 Oncology, School of Medicine, Western Sydney University, Penrith, AUS

**Keywords:** biological response modifier, chemotherapy, immunotherapy, mgn-3, immunomodulator, polysaccharide, biobran

## Abstract

Background

The effect of rice bran arabinoxylan compound (RBAC), a plant-based immunomodulator, on the quality of life (QoL) in cancer patients and underlying physiological pathways remains unclear.

Trial design

The RBAC-QoL study, a double-blind, randomised, controlled pilot feasibility study, aimed to determine RBAC’s effects on QoL and the associated action mechanisms. Primary outcomes were the EORTC QLQ-C30 functional, symptom, and global QoL scores with inflammatory, nutritional, and cytokine parameters as secondary and exploratory outcomes.

Methods

Participants were adults diagnosed with solid organ tumours (≥ stage II) undergoing active treatment in several outpatient centres in New South Wales, Australia. Interventions were RBAC or matched placebo at 3g/day for 24 weeks allocated through stratified randomisation with participants, oncologists, and data collectors blinded. Data was collected from five study visits six weeks apart. The trial remained ongoing as of December 2023. An interim intention-to-treat analysis was performed using repeated measure ANOVA with pairwise comparisons where statistical significance was observed and adjusted with covariates.

Results

Global QoL scores from currently available data (n = 16; RBAC = 7, placebo = 9) were statistically different between groups (F_1,8_ = 8.6, p = 0.019, eta^2^[g] = 0.267). Pairwise comparisons found significant differences at Week 6 (p = 0.032, Cohen’s d = 1.454) and marginally at Week 12 (p = 0.069, d = 1.427). Age-adjusted analysis showed a continuous upward trend in QoL improvement over time with RBAC, while the placebo group did not deviate from baseline QoL. Significant elevations of serum white blood cell count (Week 18) and total protein (Weeks 12 and 18) were detected in the RBAC group compared to placebo. The total protein levels correlated highly with white blood cell count (Pearson’s r = 0.539, p < 0.001) and moderately with the global QoL scores (r = 0.338, p = 0.01). No intervention-related adverse events were reported in both groups.

Conclusions

RBAC improves QoL beyond placebo during active cancer treatment, possibly through the immuno-nutritional pathway - these findings, though preliminary, are valuable for future research.

Funding and registration: Daiwa Pharmaceutical Co., Ltd, Japan; BioMedica Nutraceuticals Pty Ltd., Australia. ANZCTR Reg No: ACTRN12619000562178p.

## Introduction

Rice bran arabinoxylan compound (RBAC) is a heteropolysaccharide derivative of defatted rice bran obtained through enzymatic treatment with shiitake mushroom (*Lentinus edodes*) mycelium. First developed by Daiwa Pharmaceutical Co. Ltd. (Tokyo, Japan) as MGN-3 in the early '90s, the product has since been commercially marketed as a nutraceutical for the immune system worldwide under brand names such as BioBran (globally), Lentin Plus (Japan/Asia), Ribraxx (Australia/New Zealand), and BRM4 (United States of America). Notably, due to its immunomodulating properties, RBAC is used by some cancer patients as a complementary therapy alongside conventional oncological treatment [1].

Although the exact chemical composition of the immunologically active ingredient of RBAC remains unclear, research based on MGN-3 found RBAC to be a complex polysaccharide with arabinoxylan as its primary structure (36%) while also containing galactan and glucan [2]. In preclinical experiments with healthy cells and animals, RBAC was shown to promote innate immune defences by upregulating macrophage phagocytosis and enhancing natural killer cell activity while lowering oxidative stress through strong antioxidant capacity [2]. RBAC has also been reported to augment adaptive immunity by promoting T and B lymphocyte proliferation through the induction of dendritic cell maturation. Moreover, in other preclinical studies, RBAC inhibited mast cell degranulation during allergic reactions to lower inflammation and downregulated angiogenesis by modulating cytokines and growth factors [2].

In cancer research, RBAC has exhibited anticancer properties by arresting in vivo tumour growth in murine models and in vitro experiments showed RBAC promoted apoptosis in cancer cells by increasing the responsiveness of CD95 (Fas/APO-1) ligands [3]. RBAC has also been demonstrated to restore immune dysfunction in cancer patients by upregulating natural killer cell activity and modulating cytokine production to enhance inflammatory and cytotoxic responses [3].

A previous review by the lead authors (SLO and SCP) has found evidence to support RBAC as an immunomodulator for complementing conventional cancer treatment with favourable effects, including enhancing the immune profile, reducing side effects, improving treatment outcomes, and increasing survival rates [1]. Promising results from Tan and Flores [4] also showed that head and neck cancer patients taking RBAC during radiation treatment had significantly better health-related quality of life (QoL) than patients taking placebos. However, the impact of RBAC on the QoL of patients with different types of solid organ tumours remained unclear. Hence, there is a need for further research to validate the beneficial effect of RBAC on the QoL in cancer patients and the underlying physiological pathways. 

The RBAC-QoL study was a pilot feasibility study based on a double-blind, randomised, controlled trial (RCT) design, which aimed to determine the potential effect of RBAC compared to placebo on the QoL of cancer patients undergoing active treatment. The secondary objectives were to determine the associations between RBAC intervention and the nutritional and inflammatory status of the body as the possible mechanisms influencing the perceived QoL of the patients via the psycho-neuro-immune axis. The study also attempted to explore whether cytokine modulation and changes in the gut microbiota could be potential biological pathways for RBAC to wield its QoL effects. The present article sets forth a statistical analysis protocol for the RBAC-QoL study and presents the results of an interim analysis based on the prescribed statistical methods.

## Materials and methods

The study protocol was published for open access [5]. A synopsis of the clinical trial is presented in this article, with approved variations from the original protocol highlighted. 

Research ethics approval

The Human Research Ethics Committee of Concord Repatriation General Hospital, Sydney Local Health District (Application No. 2019/ ETH00489) and Charles Sturt University HREC (Protocol No. H19244) approved the research project. The trial has been registered with the Australian New Zealand Clinical Trials Registry (ANZCTR Reg No: ACTRN12619000562178p) before recruitment.

Study synopsis

Eligibility Criteria

Recruitment targeted cancer patients visiting outpatient cancer centres with the following inclusion criteria: adult patients aged 18 years and above at the time of providing informed consent who were diagnosed with any solid organ cancer (including but not limited to colon, breast, melanoma, lung, pancreatic, bladder, and prostate) of stage II and above, currently undergoing active cancer treatment, those who received an explanation of the purpose and methods of the study and provided written consent before the start of the trial. Consenting patients were also required to maintain adequate bone marrow, liver, and kidney functions.

Cancer patients fulfilling the inclusion criteria while having ongoing but stable conditions, such as diabetes, hypertension, and chronic obstructive pulmonary disease, were included. Furthermore, patients with autoimmune vitiligo or alopecia, stable hypothyroidism on hormone replacement, and any chronic skin condition that did not require systemic therapy were also allowed to join.

The exclusion criteria were: those with existing mental health conditions that might impede the ability to provide consent, inability to complete the QoL questionnaire with minimal assistance, pregnant, lactating, or planning to get pregnant during the period of the study, active or prior documented autoimmune or inflammatory disorders within the last five years (except if otherwise mentioned above).

Randomisation, Intervention and Participation

Participants were randomly assigned into two groups: RBAC and placebo. Both groups were drawn from the same pool of consenting participants who fulfilled the eligibility criteria based on a stratified randomisation algorithm considering metastatic status (yes or no) and treatment (chemotherapy or immunotherapy). The study interventions were either RBAC or a placebo powder with similar colour, odour, and taste (1g x 3 times per day). Both the RBAC and placebo powder were manufactured and supplied by Daiwa Pharmaceutical Co. Ltd., and the plastic sachets that contained both RBAC and placebo powder were also identical in appearance. The participant, the treating oncologist, and the study coordinator (data collector) were blinded to the actual content of the study intervention.

In the 24-week trial period, each participant attended five study visits six weeks apart starting from baseline (Week 0). The participants continued with their oncological treatment according to their prescribed regimens. At each study visit, each participant was to have body composition measurements taken on a Bioelectric Impedance Analysis scale (Tanita, Kewdale, Australia), complete a set of questionnaires and undergo blood tests. Participants who opted for gut microbiome analysis also provided stool samples.

Outcome Measures

The primary outcome measure of this study was self-reported QoL based on the European Organisation for the Research and Treatment of Cancer (EORTC) core 30-item QoL questionnaire (QLQ-C30). The QLQ-C30 is composed of both multi-item scales and single-item measures. These include five functional scales (physical, role, emotional, cognitive, and social), three symptom scales (fatigue, nausea and vomiting, and pain), a global QoL scale, and six single items (dyspnoea, insomnia, appetite loss, constipation, diarrhoea, and financial difficulties). The scoring of QLQ-C30 followed the QLQ-C30 manual stipulated by EORTC [6]. Each of the 15 scales/items was mapped into a linear transformation of 0-100. Each scale/item was an outcome variable for analysis to determine the most appropriate primary outcome measures that best reflect any potential effect of RBAC compared to placebo on the QoL of cancer patients.

The secondary outcome measures included body composition parameters (body weight, body fat ratio, and muscle mass), body mass index (BMI), neutrophil to lymphocyte ratio (NLR), and the inflammatory-nutritional index (INI = the ratio of C-Reactive Protein [CRP] and albumin). These were nutritional and inflammatory indicators of cancer patients, of which any correlations with the QoL scores might help to explain the mechanisms of RBAC as an intervention.

Exploratory outcome measures were serum cytokine profiles at each visit to assess the potential immunomodulating effects of RBAC. The original plan was to evaluate 42 human cytokine/chemokine markers with multiplexing analysis, as stated in the protocol [5]. However, with the increasing cost exacerbated by currency depreciation, the number of parameters was reduced to 15 due to budget constraints. The 15-plex analysis covered granulocyte-macrophage colony-stimulating factor (GM-CSF), interferon-gamma (IFN-γ), interleukin (IL)-1β, IL-1RA, IL-2, IL-4, IL-5, IL-6, IL-8, IL-10, IL-12p40, IL-12p70, IL-13, monocyte chemoattractant protein-1 (MCP-1), and tumour necrosis factor-alpha (TNF-α).

The present study planned to compare the microbiota diversity (alpha diversity) and composition of different gut bacteria groups (beta diversity) between the two groups by analysing the faecal microbiome based on 16S ribosomal ribonucleic acid (rRNA) gene sequencing. Thus, gut microbiome analysis was another set of exploratory outcomes to assess the gut-immune-brain axis as a potential pathway through which RBAC may influence cancer patients’ QoL.

The safety assessment was based on routine blood tests at study visits. These tests were complete blood count, liver function, electrolytes, urea, creatinine and prealbumin. The study also tracked and analysed adverse events reported by participants during clinical visits throughout the trial using the Common Terminology Criteria for Adverse Events (CTCAE) version 5.0. 

Sample Size

The recruitment target was 50 participants with equal distribution in two groups. This sample size calculation was based on repeated measures (RM) analysis of variance (ANOVA) with (two groups and five repeated measures) using a priori parameters with an alpha of 0.05, power of 0.8, and an effect size of 0.35, resulting in a total sample size of 42. The effect size estimate of 0.35 was used according to the guidelines for sample size calculations for QLQ-C30 scores [7]. The recruitment target is also consistent with sample size recommendations for a pilot study, as mentioned in the protocol [5]. 

Lifestyle Factors

The present study also collected data on the lifestyle factors identified as potential confounding variables that may affect QoL during cancer treatment. Participants completed an Australian Eating Survey® food frequency questionnaire (AES FFQ) to assess dietary patterns (Visits 0 and 4) [8], an International Physical Activity Questionnaire (IPAQ) for physical activeness (Visits 0 - 4) [9], and a Use of Complementary and Alternative Medicine Questionnaire (CAMQ) to determine usage and perceived value of complementary therapies (Visits 0, 2 and 4) [5]. However, these questionnaires were optional components, which the participants could choose to opt out of.

An Australian Recommended Food Score (ARFS) was derived from the AES FFQ to provide a diet quality index ranging from 0 to 73 to indicate the consumption level of core nutrient-dense foods, including vegetables, fruit, protein foods, bread/cereals and spreads/sauces recommended in the Australian Dietary Guidelines [8]. A higher ARFS score indicated a healthier diet. Scoring of the IPAQ was based on the protocol specified by Forde [9]. The score was reported in metabolic equivalents (MET) minutes per week, representing the amount of energy expended carrying out physical activity. The MET/week score was also mapped into three categories: low, moderate and high activity levels.

CAMQ was a custom-designed questionnaire comprising 15 items covering the usage frequency of different complementary therapies, including herbal medicine, nutritional supplements, massage, etc. The questionnaire assessed the respondent’s perception towards the efficacy of complementary therapies. A copy of the questionnaire is available for downloading online [5]. The scoring of the CAMQ questionnaire was similar to the QLQ-C30 procedure, whereby the items were grouped and transformed into a global score with a linear scale of 0-100 (CAMQ score).

Sample Collection, Preservation and Assay

At sites participating in sample collection for cytokine analysis, blood samples were collected from each participant at each study visit and centrifuged into serum before being placed in 2 x 0.6 ml microcentrifuge tubes (duplicate) for transportation and storage in a -80 ^o^C facility. This study used Luminex xMAP technology for multiplex quantification of analytes. The multiplexing analysis was performed using the Luminex™ 200 system (Luminex, Austin, TX, USA) by Eve Technologies Corp. (Calgary, AB, Canada). According to the manufacturer’s protocol, 15 markers were simultaneously measured in the samples using Eve Technologies’ Human Focused 15-Plex Discovery Assay® (MilliporeSigma, Burlington, MA, USA). The assay sensitivities of these markers ranged from 0.14 - 5.39 pg/mL. Individual analyte sensitivity values were available in the MilliporeSigma MILLIPLEX® MAP protocol [10]. The average raw fluorescence signal (FI) from duplicate testing of each sample was used in the analysis.

Stool samples were to be collected by participants using a fit-for-purpose specimen collection kit (Microba Life Sciences, Brisbane, QLD Australia) within three days of each study visit. The heat-stable faecal swabs were collected from participants and stored in a -80 ^o^C freezer. Samples were subjected to DNA extraction using the microbiome DNA isolation kit (Norgen Biotek Corp., Thorold, ON, Canada) before being sealed and transported to a microbiology laboratory for amplification and sequencing using the Illumina MiSeq platform (Illumina, Inc. San Diego, CA), targeting the 16S rRNA V3-V4 gene region of the DNA.

Protocol Variations

Several protocol variations were been approved and implemented since the publishing of the study protocol. Firstly, the study was originally a single-site study at a regional cancer centre in the Central West region of New South Wales, Australia. However, the study was expanded to include three additional outpatient cancer care centres in the Central West and Sydney regions. Due to logistic considerations, collecting serum samples for cytokine analysis was made optional by the site. Consequently, three of the four sites participated in the additional serum sample collection for cytokine analysis. Secondly, as many cancer patients visiting the study sites were elderly, the upper limit of 70 years old in the original inclusion criteria was removed to facilitate recruitment efforts without compromising safety.

Thirdly, a research contingency plan was implemented since the start of the COVID-19 pandemic (in April 2020) to allow for remote interactions between the study coordinators and participants, eliminating the need for on-site face-to-face meetings. Electronic versions of all questionnaires and case report forms were used to facilitate online access. The collections of the body composition data were outsourced to the pathology clinics performing the blood draw. Additionally, study interventions could be dispensed via postal service if needed. Due to the disruption of the COVID-19 pandemic, the trial completion date was revised to mid-2024.

Statistical analysis

General Principles

Records of each dataset were exported from the project database and loaded into Microsoft Excel 365 (Richmond, WA, USA) for data cleaning, such as removing duplicate entries, correcting errors, checking structural consistency and validating completeness. Calculations of ratios and scoring of questionnaires were performed before analysis. The statistical analysis software of choice in this study was RStudio (Posit, Boston, MA, USA), running with the latest version of the R programming language and any relevant R packages. Data analysis of the 16S rRNA sequences of the faecal samples was analysed with the QIIME2 (Quantitative Insights into Microbial Ecology software) pipeline [11].

Participation Summary and Comparisons

The participant flow report was presented in a Consolidated Standards of Reporting Trials (CONSORT) flowchart [12] showing the numbers of patients screened for eligibility, randomly assigned, completed and analysed for the primary outcome. Also, for each group, exclusions at screening and dropouts after randomisation were reported together with reasons. The number of participants who opted to provide stool samples and those who agreed to complete additional lifestyle-related questionnaires were also reported. Furthermore, the completion and dropout distribution of the two groups were tested for any nonrandom association. Moreover, the compliance rate based on the percentage of intervention sachets consumed at the end of the trial out of the total number of sachets issued to each participant was reported.

The baseline characteristics of the participants to be compared include age, sex, primary cancer, cancer stage, recurrence (Yes/No), metastasis (Yes/No) and primary treatment (chemotherapy/immunotherapy). The participant’s age was computed from the difference between baseline date (visit 0) and date of birth. 

In general, hypothesis testing between the two groups was carried out to compare participant characteristics at baseline, compliance to treatment and adverse events. Continuous variables were reported in the format of mean ± standard deviation. The difference between means of two continuous variables was analysed with two-sided Student’s t-statistics. Fisher's exact test was used to determine if there were nonrandom associations between two categorical variables. A p-value of less than or equal to 0.05 was considered statistically significant.

Primary Analysis

The RBAC group was compared against the placebo group across multiple time points for all outcome measures. The primary analysis was based on the intention-to-treat principle using every data point collected from participants in the trial regardless of withdrawal. Missing data due to dropout or administrative errors was handled with pairwise deletion to maximise all data available. 

Each outcome variable, except the gut microbiome parameter, was analysed as a dependent variable in RM ANOVA, with intervention (group) as a between-subject factor and time (study visit) as the within-subject factor. The lifestyle factors of diet, physical activity and use of complementary therapies were analysed using RM ANOVA similar to the outcome variables, with dependent variables being AFRS, MET score and CAMQ score, respectively.

Box-Cox transformation on the dependent variables and removal of extreme outliers were methods used to ensure conformity to the normality assumption of RM ANOVA. For factors that failed Mauchly's test for sphericity, the p-value of the Huynd-Feldt correction was used as the significance value for testing. The F statistics with degrees of freedom, p-value and the effect size (generalised eta^2^) were reported for significant outcomes. The effect size interpretation of eta^2^ is small (eta^2^ ≥ 0.01), medium (eta^2^ ≥ 0.06) and large (eta^2^ ≥ 0.14). Pairwise comparisons were performed where significance was observed. The Bonferroni adjustment or the false discovery rates (FDR) was applied to adjust the p-values in case of multiple comparisons to control the final type-I errors. The effect sizes of the pairwise comparisons in Cohen’s d were reported and interpreted as small (d ≥ 0.2), medium (d ≥ 0.5) and large (d ≥ 0.8). For outcome variables with significance detected, Pearson’s correlation coefficient (r) was used to detect the strengths and directions of the relationships.

Adjusted Analysis

Covariance analysis (ANCOVA) was performed if there were any significant differences between groups in any baseline characteristics of the participants or lifestyle factors. Outcome variables with significant differences detected in the primary analysis were analysed to determine any influence from the differences in the baseline characteristics or lifestyle factors as covariates. The Estimated Marginal Means (emmeans) of the outcome variables were calculated to adjust for any significant impact, with pairwise comparisons performed to predict the differences after considering all confounding factors.

Microbiome Analysis

The 16S rRNA data was loaded into the QIIME2 pipeline for preprocessing to locate amplicons, remove chimeric reads and minimise noise created by spurious operational taxonomic units using the DADA2 plugin to trim and truncate only the quality sequences for analysis. Alpha diversity was calculated using the richness of ASVs (Amplicon Sequence Variants), Chao1 index, Shannon index and Faith's phylogenetic diversity. Beta diversity was calculated using four different methods: unweighted unique fraction metric (unifrac), weighted unifrac, Bray Curtis and Jaccard. Patterns in diversity as a response to the intervention of RBAC were visualised with PCoA (Principal Coordinate Analysis) with the statistical significance of groupings validated with an ANOSIM (Analysis Of SIMilarity) test in the context of other potentially interacting variables in the dataset. Differentially abundant microbial taxa that distinguish between groups were identified using ANCOM (Analysis of Composition of Microbiomes) and further visualised with the WGCNA (Weighted Correlation Network Analysis).

## Results

The following section presents the interim analysis results of the RBAC-QoL study based on data collected from two cancer centres in the Central West region of New South Wales until January 2023. Recruitment was ongoing at the time of analysis.

Recruitment flow

**Figure 1:**
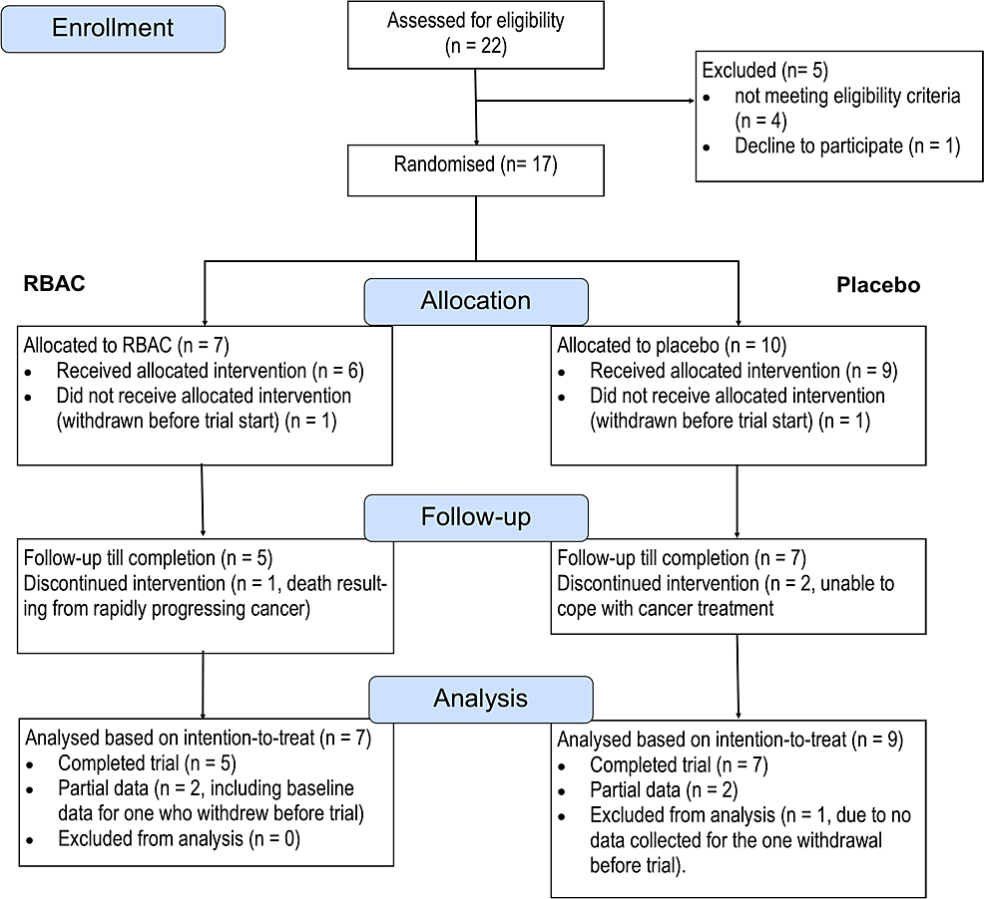
The recruitment flow of the RBAC-QoL study from June 2020 to January 2023 in a CONSORT diagram.

The recruitment flow is depicted in Figure 1.

Between June 2020 and January 2023, 22 patients consented to participate in the study and were assessed for eligibility. Five were excluded; three did not meet the criteria for maintaining adequate major organ functions. One was excluded due to exceeding the age limit (which led to the subsequent removal of the upper age limit in a protocol amendment). Another patient declined to participate after screening but before randomisation. The patient complained of being unable to cope with cancer symptoms and treatment.

A total of 17 patients were randomised into the RBAC (n = 7) and placebo group (n = 10). In the RBAC group, one participant withdrew after baseline data collection before starting the trial. Another participant in the RBAC group was hospitalised two weeks into the trial and later passed away due to rapidly progressing malignancy. The death was deemed unrelated to the study intervention. Hence, only five participants completed the study. In the placebo group, one participant withdrew immediately after screening and before baseline data collection. Two participants discontinued the trial and withdrew from the study due to not being able to cope with the cancer treatment. As such, only seven in the placebo group completed the trial.

Based on the intention-to-treat principle, all randomised participants with at least some data collected were included in the analysis. In this study, only one participant from the placebo group was excluded from data analysis, as the participant withdrew before providing any baseline data. Hence, data from 16 participants (RBAC=7, placebo=9) were analysed for the primary outcome. There is no significant difference in the ratio of completion and dropout between the two groups. Additionally, the compliance rates among the participants who completed the trial were high, with both groups achieving >99%.

Regarding the optional trial elements, only one out of seven participants in the RBAC group consented to provide faecal samples, compared to four out of nine participants in the placebo group. Only one participant in each group opted not to complete additional questionnaires for lifestyle factor assessment.

Participant characteristics

Table 1 compares the participant characteristics between the two groups. Both groups had predominantly male participants (RBAC=85.7%, placebo=88.9%), each with only one female participant. There were no significant differences in the frequency distribution of sex, trial status, cancer stage, recurrency, metastasis, and treatment types based on Fisher’s exact tests. However, the RBAC group had a mean age of 70.8 ±7.34 years, marginally different from the placebo group (64±6.70, p = 0.079). However, when tested with the alternative hypothesis that the mean age of the RBAC group is greater than that of the placebo, the one-sided p-value was statistically significant (p = 0.040). Hence, participants in the RBAC group were significantly older than those in the placebo group, making age a potential influencing factor that needs to be accounted for.

**Table 1:** Participant characteristics Continuous variable is presented in mean ± standard deviation, and the hypothesis testing of two means is based on the two-sided Student’s t-test. Significant testing of categorical variables is computed with Fisher’s exact test. RBAC=rice bran arabinoxylan compound

		RBAC	Placebo	*p*-value
N (available for analysis)		7 (100%)	9 (100%)	
Age		70.8 ±7.34	64.0 ±6.70	0.079
Sex	Male	6 (85.7%)	8 (88.9%)	1.0
	Female	1 (14.3%)	1 (11.1%)	
Trial Status	Withdrawn	1 (14.3%)	2 (22.2%)	0.758
	Deceased	1 (14.3%)	0 (0%)	
	Completed	5 (71.4%)	7 (77.8%)	
Primary Cancer	Colon and Rectal	0 (0%)	4 (44.4%)	0.177
	Melanoma	3 (42.8%)	2 (22.2%)	
	Lung	2 (28.6%)	1 (25.6%)	
	Bladder	1 (14.3%)	0 (0%)	
	Stomach	1 (14.3%)	0 (0%)	
	Oesophageal	0 (0%)	1 (11.1%)	
	Kidney	0 (0%)	1 (11.1%)	
Cancer Stage	III	2 (28.6%)	3 (33.3%)	1.0
	IV	5 (71.4%)	6 (66.7%)	
Recurrent	No	3 (42.9%)	6 (66.7%)	0.615
	Yes	4 (57.1%)	3 (33.3%)	
Metastasis	No	1 (14.3%)	1 (11.1%)	1.0
	Yes	6 (85.7%)	8 (88.9%)	
Treatment	Chemotherapy	4 (57.1%)	6 (66.7%)	1.0
	Immunotherapy	3 (42.9%)	3 (33.3%)	

As for primary cancer sites, the RBAC group included patients with malignant melanoma (n=3), lung (n=2), bladder (n=1), and stomach (n=1) cancers, whereas the placebo group consisted of patients diagnosed with cancers of the colon and rectal (n=4), malignant melanoma (n=2), lung (n=1), oesophageal (n=1), and kidney (n=1). It appeared that the placebo group was over-represented by colon and rectal cancers. However, testing for nonrandom associations between primary cancer site and group allocation was not statistically significant (p = 0.177).

Primary outcome analysis

No statistically significant difference in effects at different time points between groups was detected for all QLQ-C30 scales/items except one: the global QoL score (QL2). The QL2 score was statistically different between groups (F_1,8_ = 8.6, p = 0.019, eta^2^[g] = 0.267). However, the effects of time and the interaction between time and group were not statistically significant.

The pairwise comparisons of mean QL2 between groups for each time point are shown in Table 2. A statistically significant difference in global QoL was observed at Visit 1 (Week 6), with the RBAC group scoring 76.67 ± 6.97 in mean QL2 compared to 57.29 ± 17.50 in the placebo group (p = 0.032, Cohen’s d = 1.454). The mean QL2 difference between RBAC and placebo at Visit 2 (Week 12) was also marginally significant (79.77 ± 4.81 vs. 67.86 ± 10.13, p = 0.069, Cohen’s d = 1.427). The effect sizes at Weeks 6 and 12 were considered large.

**Table 2:** Pairwise comparisons of mean global quality of life score (QL2) between groups over time. ^ Statistically significant difference between two means based on the two-sided Student’s t-test with p < 0.05 (with multiplicity adjusted with false discovery rate). RBAC=rice bran arabinoxylan compound

Visit	Variable (unit)	RBAC	Placebo	p-value	Cohen’s d
0 (Baseline)	QL2 (1-100)	75.0 ± 23.57	63.10 ± 19.16	0.261	0.554
1 (Week 6)	QL2 (1-100)	76.67 ± 6.97	57.29 ± 17.50	0.032 ^	1.454
2 (Week 12)	QL2 (1-100)	79.77 ± 4.81	67.86 ± 10.13	0.069	1.427
3 (Week 18)	QL2 (1-100)	75.0 ± 11.79	73.81 ± 10.13	0.852	0.108
4 (Week 24)	QL2 (1-100)	81.67 ± 27.89	65.48 ± 21.75	0.172	0.647

**Figure 2:**
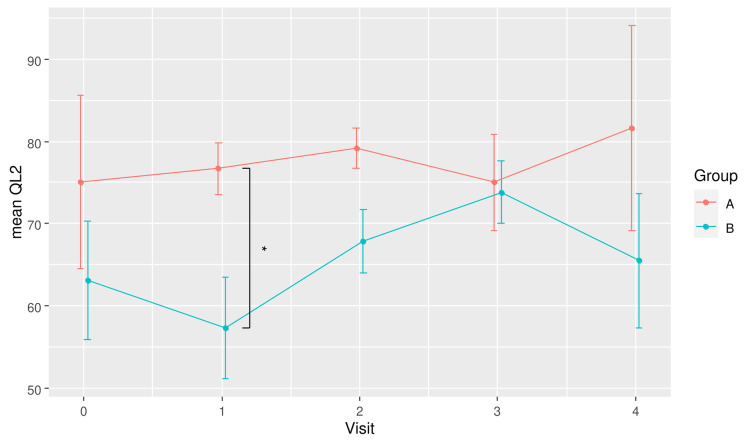
A plot showing the mean global quality of life scores (QL2) for RBAC (A) and placebo (B) groups over time. * Significant difference p < 0.05. RBAC=rice bran arabinoxylan compound

The difference in mean QL2 scores between groups over time is also depicted in Figure 2. The plot shows a dip in the global QoL of the placebo group at Visit 1 (Week 6) and recovered over the subsequent two visits before dropping again at the last visit. However, these differences over time were not statistically significant in pairwise comparison. In contrast, the global QoL of the RBAC group was relatively consistent over time. The most significant between-group difference was at Visit 1. 

Secondary outcome analysis

The body composition data collection was to be completed at pathology collection centres after the COVID-19 pandemic. However, many participants did not visit these collection centres for blood draws during the trial. As a result, body composition data, particularly body fat ratio and muscle mass, were not measured. Hence, only body weight and height from the participants’ clinical data were available for outcome analysis.

RM ANOVA was conducted on body weight, BMI, and the blood markers of NLR, and INI. No statistical significance results were detected between groups and across time points for these parameters. Therefore, the interim analysis could not isolate any potential RBAC effect on these nutritional and inflammatory markers beyond the placebo. 

Exploratory outcome analysis

Among the 15 cytokines, chemokines, and growth factors analysed, only IL12p40 showed a significant result for the main analysis based on RM ANOVA. An effect across different time points was detected (F_4, 36_ = 2.633, p = 0.05, eta^2^[g] = 0.05). However, the subsequent pairwise comparisons of different time points did not reveal any significant difference between any two visits for both groups. After eliminating extreme outliers from the dataset, the RM ANOVA analysis showed no significant change across time. Hence, the initial finding was a false positive due to a violation of the RM ANOVA assumptions caused by extreme outliners.

Microbiome analysis was not carried out in this interim analysis due to insufficient samples. Out of the five participants who agreed to provide faecal samples (RBAC=1, placebo=4), only 22 specimens were collected, with one in the placebo group dropping out after the second visit. Performing DNA amplification and sequencing using the Illumina MiSeq platform with such limited samples was not cost-effective. As such, the analysis has been deferred.

Safety outcome analysis

No safety issues were detected for all participants in their routine clinical assessment based on their blood tests of complete blood count, liver function, electrolytes, urea, creatinine and prealbumin. Two markers, white blood cell count (WBC) and total protein (TP), showed significant differences based on RM ANOVA and pairwise comparisons after multiplicity corrections. The WBC test was significantly different across time (F_4,36_ = 4.572, p = 0.004, eta^2^[g] = 0.175), whereas the TP test showed significant effects between groups (F_1,9_ = 5.133, p = 0.05, eta^2^[g] = 0.288) and the interaction of group and time (F_4,36_ = 4.137, p = 0.007, eta^2^[g] = 0.118).

Table 3 shows the pairwise comparisons of the means of WBC and TP for the two groups across different time points. Significant between-group differences were observed for WBC at Visit 3, with 7.68 ± 0.99 for RBAC, higher than the 5.80 ± 1.13 recorded in the placebo group (p = 0.022, Cohen’s d = 1.767). The mean TP values of the RBAC group were also higher than those of the placebo group at Visit 2 (77.00 ± 2.16 vs. 69.13 ± 5.03, p = 0.015, Cohen’s d = 2.035) and Visit 3 (79.50 ± 5.97 vs. 68.86 ± 4.88, p = 0.010, Cohen’s d = 1.952). Note that the difference in mean TP at Visit 4 was also marginally significant (p = 0.056, Cohen’s d = 1.421). 

**Table 3:** Pairwise comparisons of white blood cell count (WBC) and total protein (TP) between groups over time. ^ The statistically significant difference between the two means is based on the two-sided Student’s t-test with p < 0.05 (multiplicity adjusted with false discovery rate). RBAC=rice bran arabinoxylan compound

Visit	Variable (unit)	RBAC	Placebo	p-value	Cohen’s d
0 (Baseline)	WBC (x10^9^/L)	6.35 ± 2.61	5.78 ± 1.80	0.622	0.255
1 (Week 6)	WBC (x10^9^/L)	5.48 ± 2.61	5.30 ± 1.60	0.887	0.081
2 (Week 12)	WBC (x10^9^/L)	7.53 ± 1.81	5.31 ± 2.35	0.132	1.055
3 (Week 18)	WBC (x10^9^/L)	7.68 ± 0.99	5.80 ± 1.13	0.022 ^	1.767
4 (Week 24)	WBC (x10^9^/L)	8.80 ± 1.22	6.63 ± 2.45	0.137	1.122
0 (Baseline)	TP (g/L)	72.50 ± 3.15	70.33 ± 7.45	0.516	0.379
1 (Week 6)	TP (g/L)	72.50 ± 2.65	70.00 ± 5.56	0.421	0.575
2 (Week 12)	TP (g/L)	77.00 ± 2.16	69.13 ± 5.03	0.015 ^	2.035
3 (Week 18)	TP (g/L)	79.50 ± 5.97	68.86 ± 4.88	0.010 ^	1.952
4 (Week 24)	TP (g/L)	75.50 ± 3.87	69.29 ± 4.82	0.056	1.421

**Figure 3:**
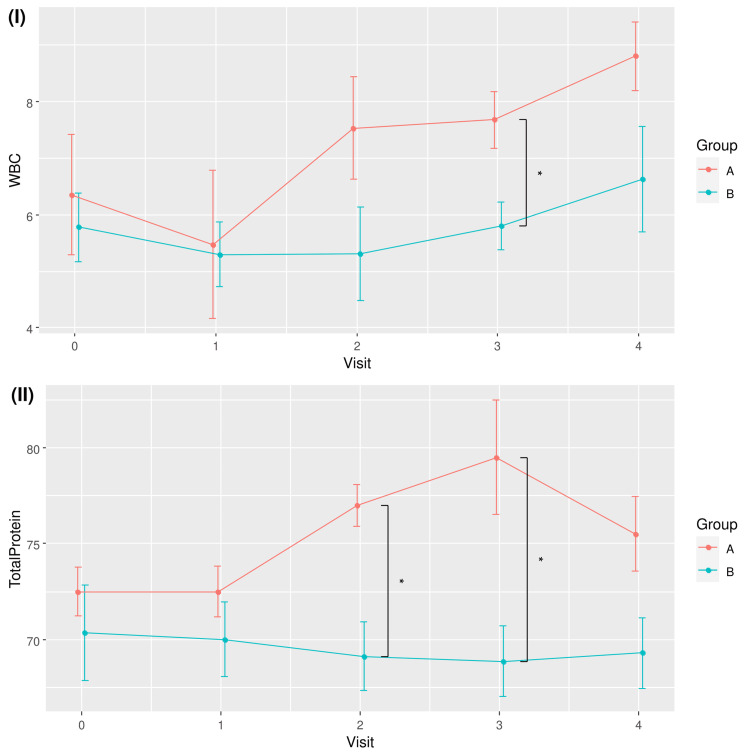
Plots of (I) mean white blood cell count (WBC) and (II) total protein for RBAC (A) and placebo (B) groups over time. * Significant difference p < 0.05. RBAC=rice bran arabinoxylan compound

The trends of WBC and TP for the two groups over time are visualised in Figure 3. Although time effects were detected as statistically significant with RM ANOVA, pairwise comparisons of different time points by groups did not yield any significant difference after the p-values were corrected for multiplicity with FDR.

Correlations of significant outcomes

The pairwise correlation coefficients of the QL2, WBC and TP as significant outcomes of this trial are shown in Table 4. A strong positive correlation was detected between TP and WBC (r = 0.539, p < 0.001) compared to a moderate positive correlation between TP and QL2 (r = 0.338, p = 0.010). In contrast, the correlation between QL2 and WBC was positive but weak (r = 0.156, p = 0.248). Hence, improved QoL in the participants appeared linearly associated with increased TP during treatment.

**Table 4:** Pairwise comparisons of the correlation among global quality of life score (QL2), white blood cell count (WBC), and total protein (TP). Pearson correlation coefficient (r): * moderate correlation; ** highly correlated.

Pearson’s r	QL2	WBC	TP
QL2	1	0.156 (p = 0.248)	0.338 (p = 0.010) *
WBC	0.156 (p = 0.248)	1	0.539 (p < 0.001) **
TP	0.338 (p = 0.010) *	0.539 (p < 0.001) **	1

Analysis of lifestyle factors

RM ANOVA was applied to analyse ARFS as the indicator of diet, MET score for physical activity and CAMQ score for usage and belief in complementary therapies. Analysis of ARFS and CAMQ scores were unremarkable. Only the MET score showed a significant difference for time effect (F_4,28_ = 5.376, p = 0.002, eta^2^[g] = 0.325). However, pairwise comparisons between time points by group did not reveal any significant difference for both groups after adjustment for multiple comparisons.

The differences in mean MET scores between time points become significant after combining the data from all participants, as shown in Table 5. Specifically, differences between Visits 1 and 2 (p = 0.046, Cohen’s d = -0.887), 1 and 3 (p = 0.040, Cohen’s d = -0.985), 2 and 4 (p = 0.033, Cohen’s d = 1.223), and 3 and 4 (p = 0.033, Cohen’s d = 1.370) were statistically significant.

**Table 5:** Pairwise comparisons of the participant’s physical activity level measured in MET/week at different visits. ^ Statistically significant difference between two means based on the two-sided Student’s t-test with p < 0.05 (with multiplicity adjusted with false discovery rate). (Hypothesis testing, H_o_: X = Y). MET=metabolic equivalent

Visit (X)	MET/Week (X)	Visit (Y)	MET/Week (Y)	p-value (X = Y)	Cohen’s d
0 (Baseline)	794.27 ± 344.78	1 (Week 6)	788.29 ± 681.87	0.391	0.011
		2 (Week 12)	1473.60 ± 853.55	0.162	-1.044
		3 (Week 18)	1489.50 ± 740.87	0.119	-1.203
		4 (Week 24)	594.64 ± 166.50	0.153	0.434
1 (Week 6)	788.29 ± 681.87	2 (Week 12)	1473.60 ± 853.55	0.046 ^	-0.887
		3 (Week 18)	1489.50 ± 740.87	0.040 ^	-0.985
		4 (Week 24)	594.64 ± 166.50	0.646	0.312
2 (Week 12)	1473.60 ± 853.55	3 (Week 18)	1489.50 ± 740.87	0.871	-0.020
		4 (Week 24)	594.64 ± 166.50	0.033 ^	1.223
3 (Week 18)	1489.50 ± 740.87	4 (Week 24)	594.64 ± 166.50	0.033 ^	1.370

**Figure 4:**
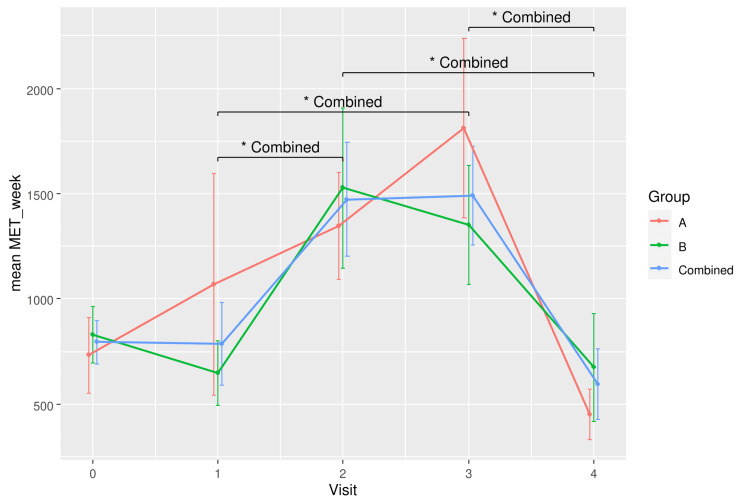
A plot showing the physical activity levels (MET/week) for RBAC (A) and placebo (B) groups and all participants combined over time. * Significant difference p < 0.05. MET=metabolic equivalent; RBAC=rice bran arabinoxylan compound

Changes in physical activity levels over time are illustrated in Figure 4. The participants were sedentary at the start of treatment (Weeks 0 to 6) but became more active physically at Weeks 12 and 18 while slowing down at the end of their treatment (Week 24). Both groups exhibited similar behaviours with no significant difference in MET score at any time point. Hence, physical activity level was not likely a confounding variable that could influence the between-group differences in the current experiment. 

Adjusted analysis

ANCOVA was performed for outcome variables QL2, WBC, and TP with age as a covariate. For the global QoL scores, the group effect was reduced after adjusting for age variation but remained marginally significant (F_1,7_ = 4.731, p = 0.066, eta^2^[g] = 0.217). The effects of time (F_4,28_ = 2.786, p = 0.046, eta^2^[g] = 0.190) and the interaction of time and age (F_4,28_ = 2.718, p = 0.050, eta^2^[g] = 0.186) were statistically significant. The emmeans predicting the QL2 values after adjusting for age are shown in Table 6. There was a significant difference between RBAC and placebo groups at Visit 3 (85.76 ± 10.42 vs. 67.66 ± 9.35, p = 0.039, d = 4.069).

**Table 6:** Pairwise comparisons of the estimated marginal means (emmeans) of global quality of life score (QL2) between groups over time after adjusting for age as a covariate. ^ Statistically significant difference between two means based on the two-sided Student’s t-test with p<0.05 (with multiplicity adjusted with false discovery rate). RBAC=rice bran arabinoxylan compound

Visit	Emmean (unit)	RBAC	Placebo	p-value	Cohen’s d
0 (Baseline)	QL2 (1-100)	64.77 ± 26.99	70.40 ± 25.28	0.762	-0.518
1 (Week 6)	QL2 (1-100)	73.51 ± 17.31	59.27 ± 16.42	0.211	2.082
2 (Week 12)	QL2 (1-100)	76.06 ± 12.09	69.63 ± 10.85	0.471	1.246
3 (Week 18)	QL2 (1-100)	85.76 ± 10.42	67.66 ± 9.35	0.039 ^	4.069
4 (Week 24)	QL2 (1-100)	89.67 ± 26.81	59.76 ± 25.88	0.112	2.733

**Figure 5:**
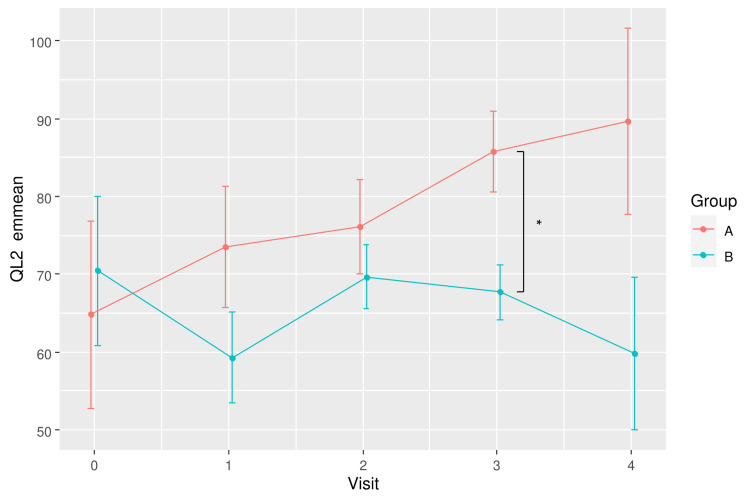
A plot showing the trends of estimated marginal means (emmeans) in global quality of life score (QL2) for RBAC (A) and placebo (B) groups over time. * Significant difference p < 0.05. RBAC=rice bran arabinoxylan compound

The predicted trends of QoL change for the two groups based on emmeans are shown in Figure 5. The RBAC group had a continuous upward trend in QoL improvement over time, whereas the placebo group showed a fluctuating QoL that did not deviate much from the baseline level.

Under ANCOVA with age as a covariate, a significant group-time interaction effect was detected for both WBC (F_4,32_ = 2.749, p = 0.045, eta^2^[g] = 0.118) and TP (F_4,32_ = 5.688, p = 0.001, eta^2^[g] = 0.147) after sphericity corrections. The emmeans of WBC and TP are shown in Table 7. A very significant between-group difference was detected after 18 weeks for WBC (8.65 ± 1.18 vs. 5.24 ± 1.06, p = 0.003, d = 6.775). For TP, significant between-group differences were shown at Week 12 (79.45 ± 5.96 vs. 67.90 ± 5.19, p = 0.019, d = 4.670) and Week 18 (81.77 ± 7.18 vs. 67.56 ± 6.44, p = 0.023, d = 4.636).

**Table 7:** Pairwise comparisons of the emmeans of white blood cell count (WBC) and total protein (TP) between groups with age as a covariate over time. ^ The statistically significant difference between the two means is based on the two-sided Student’s t-test with p < 0.05 (multiplicity adjusted with false discovery rate) and ^^ p < 0.01. RBAC=rice bran arabinoxylan compound

Visit	Emmean (unit)	RBAC	Placebo	*p*-value	Cohen’s *d*
0 (Baseline)	WBC (x10^9^/L)	6.07 ± 2.50	5.96 ± 2.40	0.940	0.119
1 (Week 6)	WBC (x10^9^/L)	5.82 ± 2.84	5.13 ± 2.48	0.728	0.590
2 (Week 12)	WBC (x10^9^/L)	7.85 ± 3.21	5.15 ± 2.80	0.249	2.024
3 (Week 18)	WBC (x10^9^/L)	8.65 ± 1.18	5.24 ± 1.06	0.003 ^^	6.775
4 (Week 24)	WBC (x10^9^/L)	9.39 ± 2.98	6.29 ± 2.67	0.177	2.436
0 (Baseline)	Neutrophils (x10^9^/L)	3.50 ± 1.54	3.68 ± 1.48	0.832	-0.332
1 (Week 6)	Neutrophils (x10^9^/L)	2.67 ± 1.84	3.03 ± 1.60	0.774	-0.487
2 (Week 12)	Neutrophils (x10^9^/L)	4.14 ± 2.12	3.21 ± 1.84	0.538	1.052
3 (Week 18)	Neutrophils (x10^9^/L)	4.75 ± 0.61	3.22 ± 0.55	0.007 ^^	5.869
4 (Week 24)	Neutrophils (x10^9^/L)	5.57 ± 2.78	4.12 ± 2.49	0.477	1.227
0 (Baseline)	TP (g/L)	71.43 ± 7.08	71.04 ± 6.82	0.924	0.150
1 (Week 6)	TP (g/L)	73.22 ± 7.10	69.64 ± 6.19	0.479	1.215
2 (Week 12)	TP (g/L)	79.45 ± 5.96	67.90 ± 5.19	0.019 ^	4.670
3 (Week 18)	TP (g/L)	81.77 ± 7.18	67.56 ± 6.44	0.023 ^	4.636
4 (Week 24)	TP (g/L)	77.21 ± 6.24	68.30 ± 5.60	0.077	3.341

**Figure 6:**
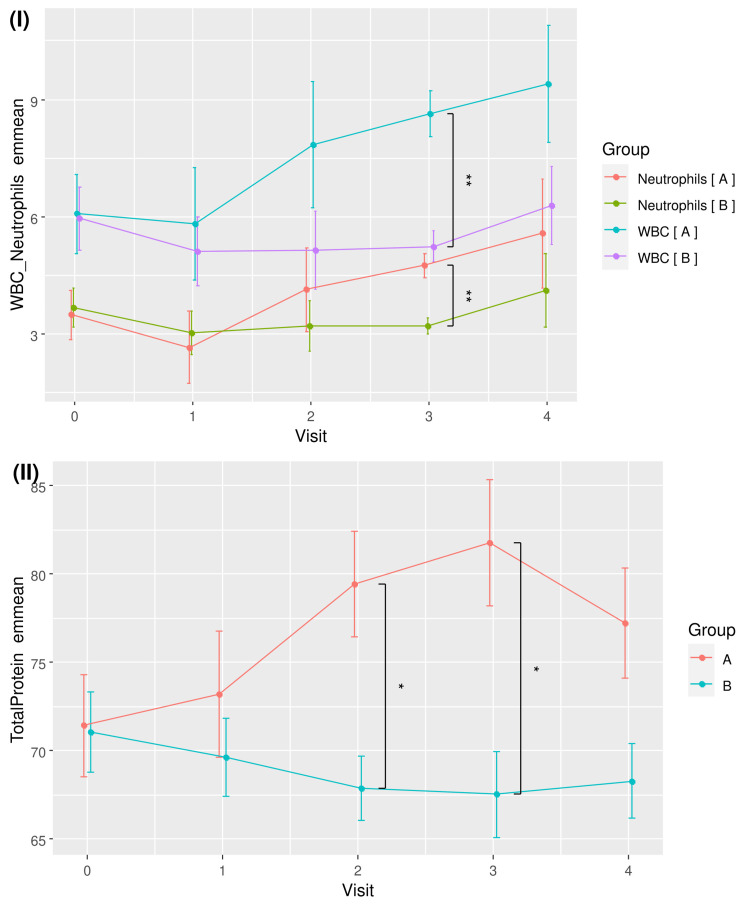
Plots of (I) emmeans of white blood cell count (WBC) plus neutrophil count and (II) total protein for RBAC (A) and placebo (B) groups over time. * Significant difference *p* < 0.05; ** *p* < 0.01.

Table 7 also includes the emmeans of the neutrophil counts of the RBAC and placebo groups. Similar to WBC, a significant between-group difference was detected for neutrophils at week 18 (4.75 ± 0.61 vs. 3.22 ± 0.55, p = 0.007, d = 5.869), albeit no effect was detected under ANCOVA. The plots of emmeans over time for WBC and neutrophils showing parallel trends for both groups are shown in Figure 6 (I). As neutrophils are the most abundant WBC, their increase during the trial set the WBC emmeans of the RBAC group higher than the placebo group over time. Figure 6 (II) plots TP emmeans over time comparing RBAC to the placebo groups. The graph shows a similar trend compared to the unadjusted mean TP values in Figure 3.

Adverse events

Table 8 shows a comparison between the two groups in terms of the adverse events reported during the trial period. The two groups had no statistically significant differences in mean adverse events reported per participant. There was one incident of life-threatening bowel obstruction in the placebo group where the patient was hospitalised. The event was resolved and deemed unlikely to be study-related. In the RBAC group, one death resulted from complications from a fast-growing malignancy unrelated to the study intervention two weeks after starting the trial. Most adverse events were mild (RBCA = 73.1%, placebo = 90%) to moderate (RBCA = 23.1%, placebo = 8%). The differences in grading distribution showed a higher portion of moderate adverse events in the RBAC group but were not statistically significant.

**Table 8:** A comparison of the adverse events reported between RBAC and placebo groups. Continuous variable is presented in mean ± standard deviation, and the hypothesis testing of two means is based on the two-sided Student’s t-test. Significant testing of categorical variables is computed with Fisher’s exact test. AE=adverse events; RBAC=rice bran arabinoxylan compound

		RBAC	Placebo	p-value
Number of Participants (N)		7	9	
Number of AE reported		26 (100%)	50 (100%)	
Mean AE per patient		3.71 ± 3.64	5.56 ± 2.92	0.2921
Grade	1 – Mild	19 (73.1%)	45 (90.0%)	0.0647
	2 – Moderate	6 (23.1%)	4 (8.0%)	
	3 – Severe	0 (0%)	0 (0%)	
	4 – Life-threatening	0 (0%)	1 (3.8%)	
	5 – Death	1 (3.8%)	0 (0%)	
Trial	1 – Not related	13 (50.0%)	28 (56.0%)	0.8314
Relationship	2 – Unlikely	12 (46.2%)	20 (40.0%)	
	3 – Possible	1 (3.8%)	2 (4.0%)	
	4 – Probable	0 (0%)	0 (0%)	
	5 – Definite	0 (0%)	0 (0%)	
Most Common	Fatigue	4 (15.4%)	7 (14.0%)	0.2529
Events	Oral Thrush	0 (0%)	5 (10.0%)	
	Diarrhoea	2 (7.7%)	3 (6.0%)	
	Peripheral neuropathy	1 (3.9%)	3 (6.0%)	
	Constipation	1 (3.9%)	3 (6.0%)	
	Cough	2 (7.7%)	2 (4.0%)	
	Nausea	2 (7.7%)	2 (4.0%)	
	Rash	0 (0%)	3 (6.0%)	
	Shortness of breath	1 (3.9%)	2 (4.0%)	
	Dysgeusia	1 (3.9%)	1 (2.0%)	
	Other isolated events	12 (46.1%)	19 (38.0%)	

Fatigue was the most common adverse event reported in both groups (RBAC = 15.4%, placebo = 14%). Participants in the placebo group reported five incidents of oral thrush, but none was reported in the RBAC group. Other adverse events reported include diarrhoea, chemotherapy-induced peripheral neuropathy, constipation, cough, nausea, rash, shortness of breath, and dysgeusia. Of all the events, 96% were deemed unrelated to the study intervention. The oncologists rated three adverse events (diarrhoea, abdominal pain, and dysgeusia) as possibly study-related, with one in the RBAC group and two in the placebo group. However, these adverse events were also commonly associated with oncological treatment. Overall, there was no significant difference between groups in adverse events reported.

## Discussion

The RBAC-QoL study aims to determine the potential effect of RBAC compared to placebo on the QoL of cancer patients undergoing active treatment. This interim analysis showed encouraging results, with RBAC showing a statistically significant difference in increasing QoL over placebo during the trial with a large effect size in the primary analysis (F_1,8_ = 8.6, p = 0.019, eta^2^[g] = 0.267). Specifically, the global QoL scores of the patients taking RBAC were higher than those taking placebos at Week 6 and marginally higher at Week 12 during the trial. The placebo group had a drop in global QoL at Week 6 before experiencing some recovery after that (Figure 2). Since the participants were starting active treatment at baseline, a decline in QoL was expected since antitumour treatment, especially chemotherapy with unwanted side effects, could negatively affect their well-being [13, 14]. The RBAC group, however, maintained their QoL throughout the trial while on active treatment, revealing the effectiveness of RBAC in QoL maintenance. Notably, the reported effect size (eta^2^[g] = 0.267) is considered large for ANOVA [15] and Cohen’s d measuring the differences in mean QL2 between groups at Weeks 6 and 12 were large and thus clinically significant [7]. The treatment effects of RBAC appeared more pronounced after adjusting for the age differences between groups (Figure 5). Such favourable observation is thus consistent with the results of Tan and Flores [4], whereby there was a statistically significant difference in the mean QoL scores (p = 0.019) between the RBAC and placebo groups two months after radiation treatment for participants with head and neck cancers. Although not explicitly accounted for in the present study, some participants also received radiation treatment in addition to chemotherapy and immunotherapy.

Other RCTs have reported RBAC’s benefits in reducing the side effects of cancer treatment. Masood et al. [16] studied the incidents of side effects in breast cancer patients taking RBAC compared to those who did not during six cycles of chemotherapy using a self-reporting questionnaire. Reductions in the proportions of patients experiencing anorexia/tiredness (RBAC vs. control: 20% vs. 88%), nausea/vomiting (40% vs. 100%), hair loss (28% vs. 100%) and weight loss (0% vs. 84%) were reported. Another study by Petrovics et al. [17] also reported significant alleviation of fatigue symptoms (p<0.001) in cancer patients with chronic fatigue syndrome after 24 weeks of RBAC plus oncothermia interventions during active treatment compared to control patients who received only conventional oncological treatment. The fatigue measurement was based on the Chalder fatigue questionnaire and Patients’ Global Impression of Change (PGIC) scales.

In the present study, fatigue was also one of the treatment side effects experienced by the participants. However, no statistically significant differences between the groups were detected in fatigue and other treatment side-effects, such as nausea and vomiting or pain, based on QLQ-C30. Such discrepancy could be due to the lack of sensitivity in the symptom scores of QLQ-C30, based on four-point Likert scales. Finstad [18] found that seven-point Likert items could provide a more accurate measure of a participant’s evaluation than a five-point scale (let alone four) in electronic questionnaires. Moreover, the lack of sensitivity in the symptom scales could also be compounded by the small sample size in this interim analysis, which may not have enough power. Therefore, to assess treatment side effects, future studies should consider augmenting the QLQ-C30 with a more specific self-evaluated adverse effects questionnaire, such as the 10-item instrument proposed by Montemurro et al. [19] and the Chalder fatigue questionnaire.

While RBAC was hypothesised to impact the QoL of cancer patients via the inflammatory and nutritional pathways, none of the secondary and exploratory outcome measures, including BMI, INI, NLR and cytokine profile, showed significant differences between groups. Notwithstanding, this interim analysis detected substantial between-group differences in WBC and TP from the routine safety assessment of participants with considerably large effect sizes, albeit not being the pre-established outcome measures of interest. The analysis found relatively higher WBC and TP in the RBAC group compared to the placebo group at Visits 2 and 3 (Figure 3). In particular, the rise in WBC resulted from rising neutrophil count in the RBAC group based on the age-adjusted analysis (Figure 6).

Two other RBAC RCTs also examined WBC. Itoh et al. [20] compared RBAC to placebo in patients with cervical cancer undergoing chemoradiotherapy. Depletion of WBC was reported in all patients after three weeks of treatment due to radiation side effects. However, no significant difference between groups was found, although the study authors commented that the control group tended to have lower WBC than the RBAC group. Radiation therapy also caused head and neck cancer patients to have lower posttreatment WBC in the RCT by Tan and Flores [4]. Similarly, there was no significant difference between the RBAC and placebo groups in this study. Therefore, RBAC was not known to affect WBC. The current study is the first to demonstrate that RBAC could potentially preserve and improve the WBC profiles of cancer patients during active treatment with chemotherapy or immunotherapy.

The potential effects of RBAC on cancer patients’ neutrophil count were previously reported by Golombick et al. [21] in a single-arm before and after study in patients with monoclonal gammopathy of undetermined significance (MGUS)/smouldering multiple myeloma (SMM). Combining RBAC with curcumin improved the neutrophil count of eight out of 10 MGUS/SMM patients after six months. In another case series by Tsunekawa [22] with 16 cancer patients who had recently completed oncological treatment, the proportion of patients who had increased, unchanged and decreased neutrophil categorisation after six months of RBAC treatment were 5/16, 5/16 and 6/16, respectively. Hence, RBAC appeared to maintain or increase the neutrophil count in 10/16 of these patients. Many cancer patients develop neutropenia due to chemotherapy, thus increasing the risk of infection [23]. Hence, the findings of this interim analysis are consistent with these earlier reports. RBAC’s immunomodulatory effect appeared to help maintain adequate neutrophil count in cancer patients during and after treatment.

TP measures the concentration of proteins dissolved in the plasma, which consists mainly of albumins and globulins [24]. Older cancer patients, those with gastrointestinal cancer and malnutrition were found to be significantly associated with hypoproteinemia [25]. As a marker for protein-energy malnutrition, TP has been found to correlate well with nutritional screening based on the subjective global assessment tool in cancer patients [25].

The current analysis found that the participants’ TP strongly correlated with their WBC level (r = 0.539) and a moderate correlation with their QoL scores (r = 0.338) was also detected. The RBAC group demonstrated better QoL at Weeks 6 and 12 of active treatment than the placebo group, coinciding with their higher TP and WBC from 12 to 18 weeks into treatment. Thus, favourable subjective patient-reported QoL outcomes precede the quantitative nutritional and immune profile measurements. These preliminary findings support that RBAC could improve QoL via the immuno-nutrition pathway by interacting with the immune system and nutritional status. However, the lower TP levels in the placebo group may also result from the overrepresentation of colorectal cancer patients with an increased risk of malnutrition [26]. There could also be other unknown factors influencing the changes in TP and WBC, given the effects appeared to be delayed. Therefore, further validation in the final analysis with a larger sample size and a more balanced group is needed.

Physical activity is recommended for all cancer patients at all stages of their treatment [27] and many oncologists routinely advise patients to be physically active [28]. The pattern of changing physical activity levels among the participants in this analysis is intriguing, albeit not directly related to the study objectives. The inverted V curve (Figure 4) depicted a scenario in which the generally sedentary participants at baseline complied with the advice and were more physically active during the treatment. However, they appeared to be lowering their physical activity level near the end of the treatment. Such behaviour warrants further investigation to inform the design of interventional exercise programmes as supportive care for cancer patients during and after treatment to improve outcomes and QoL.

Despite the favourable findings, this study has several limitations. This initial analysis is based on the results of a relatively small number of participants and thus should be interpreted with caution. A small study tends to have imprecise effect estimates and cannot be relied upon to draw firm conclusions [29]. Even with the trial reaching the targeted sample size of 50, this remains a pilot study intended to inform a future more extensive investigation. Hence, no claims of treatment efficacies are made. A second limitation is the duration of the study. Although a trial period of six months is considered sufficient to evaluate the changes in QoL during active treatment, the study does not include any follow-up to assess the longer-term effects of RBAC versus placebo at posttreatment. A third limitation is the lack of outcome measures correlating participant QoL changes with oncological treatment outcomes. Therefore, it is unclear whether the improvement in QoL could influence the effectiveness of the active treatment or survival outcomes of the patients. 

## Conclusions

This paper presents an interim analysis of the RBAC-QoL study. The preliminary findings based on 16 participants showed encouraging results that RBAC could improve the QoL of cancer patients beyond placebo during active treatment, most prominently at six to 12 weeks into the trial. The QoL improvement may be due to effects on the immuno-nutritional pathway, with the RBAC group showing higher WBC and TP levels than the placebo group, which correlated with the QoL scores. Such preliminary findings, however, should be interpreted with caution as the trial remains ongoing, and the detected effect size will likely change with a larger sample size. Regardless, this analysis provides valuable information for designing a larger RCT to confirm RBAC’s beneficial effects on cancer patients' QoL.
